# Multiple Myeloma Unpacked

**DOI:** 10.1002/hon.70067

**Published:** 2025-06-15

**Authors:** Francesca Gay, Edoardo Marchetti, Giuseppe Bertuglia

**Affiliations:** ^1^ Division of Hematology AOU Città della Salute e della Scienza Department of Molecular Biotechnology and Health Science University of Torino Torino Italy

**Keywords:** immunotherapies, myeloma, response assessment, staging

## Abstract

Multiple myeloma (MM) is a hematologic malignancy characterized by the clonal proliferation of plasma cells in the bone marrow. The current treatment landscape for multiple myeloma involves a combination of proteasome inhibitors, immunomodulatory drugs, monoclonal antibodies, and autologous stem cell transplantation, which have significantly improved survival outcomes in recent years. However, the disease remains challenging, particularly in relapsed and refractory cases. Ongoing clinical trials are evaluating new therapeutic options, including chimeric antigen receptor T cell therapy, bispecific antibodies, antibody drug conjugate and novel agents like Cereblon E3 Ligase Modulatory Drugs. This review summarizes the key diagnostic criteria, prognostic features, response assessment for MM and highlights the current treatment landscape.

## 1 MGUS and SMM: Risk Stratification and Screening Program

Multiple myeloma (MM) is a hematological malignancy characterized by the uncontrolled proliferation of tumoral plasma cells and the presence of a monoclonal immunoglobulin protein. It accounts for approximately 10% of all hematological cancers and has an increasing global incidence [1]. The disease is highly heterogeneous and genetically complex, evolving through a multistep process that includes genetic mutations in plasma cells and alterations in the bone marrow microenvironment [2]. MM typically progresses from an asymptomatic precursor stage known as monoclonal gammopathy of undetermined significance (MGUS), characterized by < 10% bone marrow plasma cells (BMPCs), observed in roughly 5% of individuals over 50 years of age. MGUS has a progression rate of 1% annually to MM or related disorders and is more common in Black individuals. Patients transition to an intermediate stage called smoldering multiple myeloma (SMM), requiring ≥ 10% BMPCs and/or serum monoclonal protein (M) ≥ 3 g/dL, without end‐organ damage [3]. The International Myeloma Working Group (IMWG) 2/20/20 model allows separation into four groups of patients with different risk of progression to symptomatic disease based on BMPCs ≥ 20%, M‐protein ≥ 2 g/dL, a free light chain ratio ≥ 20 and presence of cytogenetic abnormalities—specifically t(4; 14), t(14; 16), +1q, and/or del13q. Low risk patients with 0 risk factors have a 6% risk of progression at 2 years, low intermediate risk with 1 risk factor 23%, intermediate risk with 2 risk factors 46%, and high risk with ≥ 3 risk factors 63% [4]. The detection of an evolving pattern in serum M‐protein through non‐invasive sequential measurements may improve risk stratification [5]. There is no validated median time interval between patients' visits, but a broader consensus suggests follow‐up every 2–6 months, depending on patient risk [6].

The utility of a screening program in MM has not been established, but screening an entire country for myeloma precursors could be a bold initiative to gain deeper insights into the origins of the disease and prevent progression. In this view, the iStopMM program is a research project started in 2016 with the aim of screening the population of Iceland for the presence of MGUS or SMM. Active screening identified a number of individuals with full‐blown malignancy and smoldering disease, revealing the potential role of screening for early therapeutic interventions or more intensive monitoring [7]. The same study found that a free light‐chain (FLC) ratio of 1.65–3.15 in light chain (LC)‐MGUS, defined by FLC levels outside the reference range, was associated with no progression to advanced plasma cell disorders, while ∼6% of patients with FLC ratios > 3.15 progressed over approximately 5 years. Adjusting the FLC ratio threshold could help to prioritize monitoring for individuals at higher risk of malignancy [8].

## 2 Diagnostic and Prognostic Criteria: Changes Over Time

The IMWG revised criteria for diagnosing MM require the presence of clonal plasma cells in the bone marrow or a biopsy‐confirmed plasmacytoma, alongside with traditional CRAB features—hypercalcemia, renal failure, anemia, or lytic bone lesions—or specific biomarkers of imminent organ damage (SLiM), as ≥ 60% BMPCs, a FLC ratio ≥ 100 with elevated involved FLC levels (≥ 100 mg/L), or more than one focal lesion on magnetic resonance imaging (MRI) ≥ 5 mm [1]. Diagnosis involves quantification of serum protein electrophoresis, serum immunofixation electrophoresis, and FLC assays to detect monoclonal proteins, although about 2% of patients present with non‐secretory disease [9]. Bone marrow studies, including fluorescence in situ hybridization (FISH), identify cytogenetic abnormalities that aid in risk stratification, imaging modalities such as whole‐body low‐dose computed tomography (WBLDCT), positron emission tomography (PET/CT), and WB‐MRI assess bone lesions or extramedullary disease (EMD). Preliminary data highlight that WB‐MRI is superior in detecting diffuse and micronodular patterns in MM and has higher sensitivity in detecting focal lesions compared to PET/CT, assessing high‐risk SMM and detecting early symptomatic MM [10].

**FIGURE 1:**
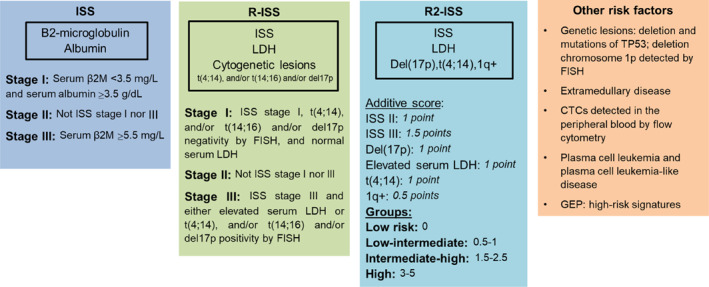
Staging system for high‐risk multiple myeloma. ISS, International Staging System; R‐ISS, Revised International Staging System; β2M, Beta‐2 microglobulin; LDH, lactate dehydrogenase; FISH, fluorescence in situ hybridization; CTCs, circulating tumor cells; GEP, gene‐expression profile.

The prognostic assessment of MM has evolved significantly over time, reflecting advances in understanding the disease and its biological heterogeneity. Initially, the Durie and Salmon (DS) staging system provided risk stratification based on tumor burden, renal function, and calcium levels [11]. Later, the International Staging System (ISS) [12] introduced serum beta‐2 microglobulin (B2M) (< 3.5 mg/L or > 5.5 mg/L) and albumin (> 3.5 g/dL) as key prognostic markers. With the advent of genomic insights, the Revised ISS (R‐ISS) incorporated chromosomal abnormalities t(4; 14), t(14; 16), del(17p) and lactate dehydrogenase (LDH) levels to further enhance risk prediction [13]. The second revision of ISS (R2‐ISS) integrating additional molecular features to further refine stratification (gain/amp 1q) was proposed at a later time [14] (Figure 1).

More recently, the International Myeloma Society (IMS), have pointed out the role of different genetic abnormalities in defining prognosis alongside with the role of B2M (under publication). Risk definition is indeed extremely complex, and several factors not included in any of the above risk stratification, can play a role and need to be considered. This is particularly true for EMD disease [15, 16], and to the presence of circulation tumor cells (CTCs) levels [17, 18].

Additionally, some patients without apparent risk factors exhibit aggressive behavior, with suboptimal responses and early relapses, defining the so‐called functional high‐risk category [19].

## 3 Highlights in Response Criteria

The IMWG response criteria define benchmarks for disease control. Monthly monitoring of measurable M protein levels in serum and urine is standard during treatment, while FLC assays are essential for non‐measurable cases [20]. Routine 24‐h urine assessments have recently been shown to have limited prognostic utility and are seldom employed in real‐world practice due to challenges with collection and storage. Except in specific contexts like AL amyloidosis or LC‐MM, monitoring serum FLC alone may be a more practical alternative, enabling more frequent testing and better tracking of disease progression [21, 22].

Measurable residual disease (MRD) evaluation using next generation flow cytometry (NGF) or next‐generation sequencing (NGS), respectively called Flow MRD and Sequencing MRD, with high sensitivity (at least one in 10^5^ cells) provides detection of residual BMPC. Sustained MRD‐negativity requires confirmed MRD‐negativity in bone marrow in two samples at least 1 year apart. Imaging‐positive MRD‐negativity combines NGF/NGS with PET/CT clearance of all tracer uptake found at baseline or reduction below mediastinal blood pool SUV [20]. Indeed, recent data showed that not only PET‐CT but also DWMRI evaluation after treatment may complement bone marrow MRD [23, 24, 25].

Although MRD‐negativity does not entirely prevent relapses, it serves as a critical prognostic marker and may potentially guide treatment strategies, as it is associated with improved progression‐free survival (PFS) and overall survival (OS) [26].

## 4 Treatment: Current Landscape and Future Directions

### 4.1 Smoldering Multiple Myeloma

The current standard of care for patients with SMM remains “watch and wait.” However, there is an ongoing debate on whether early treatment provides a clinical benefit, particularly for high‐risk SMM. Two clinical trials have already demonstrated the advantage of using lenalidomide alone (R) [27] or in combination with dexamethasone (Rd) [28, 29] over observation in patients with high‐risk SMM. Several other phase II studies have explored the efficacy of triplet regimens incorporating Rd as a backbone, combined with agents such as elotuzumab [30], ixazomib [31], or carfilzomib [32] as well as quadruplet regimen including daratumumab and carfilzomib [33]. Recently, the phase 3 randomized AQUILA study, which compared daratumumab monotherapy (up to 39 cycles/26 months) with active monitoring in patients with high‐risk SMM, demonstrated that daratumumab reduced the risk of progression to symptomatic MM or death by 51% compared to active monitoring, with good tolerability (grade 3–4 infections: 16% vs. 6%) [34].

### 4.2 First Line Setting

A major determinant of the treatment choice is still eligibility for autologous stem cell transplant (ASCT). High‐dose chemotherapy followed by ASCT is typically reserved for young and fit patients. Patients are treated with 3–4 cycles of induction therapy prior to stem cell harvest. After harvest, frontline ASCT is still recommended in Europe.

The landscape of induction treatment has evolved with the incorporation of the anti‐CD38 monoclonal antibody daratumumab (D) into the triplet bortezomib‐thalidomide‐dexamethasone (VTd) and, more recently, bortezomib‐lenalidomide‐dexamethasone (VRd). In the phase III CASSIOPEIA study, MRD‐negativity, PFS, and OS were superior in the DVTd arm compared to VTd [35, 36]; similarly, the PERSEUS study [37] showed superior MRD‐negativity and PFS in DVRd compared to VRd.

Indeed, other regimens including either, isatuximab, another anti‐CD38 monoclonal antibody, and/or carfilzomib, a second‐generation proteasome inhibitor (PI), are currently under investigation, with promising preliminary results. In part 1 of the GMMG‐HD7 trial, isatuximab‐lenalidomide‐bortezomib‐dexamethasone (Isa‐VRd) induction demonstrated superiority over VRd [38]. Additionally, the phase III IsKia trial showed superior MRD negativity with isatuximab‐carfilzomib‐lenalidomide‐dexamethasone (Isa‐KRd) as pre‐ASCT induction and post‐ASCT consolidation compared with KRd [39].

Post‐transplant maintenance therapy with R until progression or intolerance has long been the standard of care. The addition of daratumumab (DR), based on the results of the PERSEUS study, will likely be the next standard in Europe [37]. The AURIGA trial also demonstrated that DR maintenance improved the MRD‐negative conversion rate in patients who were MRD‐positive after transplant, compared to lenalidomide maintenance alone, despite the requirement for patients to be anti‐CD38 naïve at enrollment limits the applicability of these results in the current treatment landscape [40]. Another emerging debate focuses on the potential discontinuation of treatment in MRD‐negative patients, particularly if patients are standard risk. There are now some reports showing low rates of disease recurrence in MRD negative patients [41, 42, 43, 44]. Trials evaluating prospectively the issue of treatment discontinuation during maintenance are ongoing.

In transplant‐ineligible patients, VRd [45], daratumumab‐lenalidomide‐dexamethasone (DRd) [46, 47] and daratumumab‐bortezomib‐melphalan‐prednisone (DVMP) [48, 49] have been the standards of cares for years.

Recently, three large phase III trials have investigated quadruplet‐based therapy versus triplets. The FDA approval of isatuximab‐bortezomib‐lenalidomide‐dexamethasone (Isa‐VRd), based on the results of the IMROZ study [38], which demonstrated the superiority of Isa‐VRd over VRd in terms of MRD negativity and PFS, introduces a new SoC. A second randomized study, BENEFIT, compared Isa‐VRd, with a bortezomib‐modified schedule, versus Isa‐Rd, and demonstrated improved MRD‐negativity with the quadruplet regimen [50].

A third study, CEPHEUS compared daratumumab plus VRd with VRd alone, showing again improved PFS and MRD with quadruplet regimen [51]. It should be noted that all the 3 studies enrolled patients up to 80 years old with a good performance status, raising concerns about the real‐world applicability of these findings for frailer patients.

The IFM‐2017‐03 trial enrolled frail patients and compared DR with R plus low‐dose dexamethasone (Rd), investigating whether dexamethasone could be omitted in frail patients in the anti‐CD38 mAb era. Interim results suggest comparable safety but a 49% reduction in the risk of progression (mPFS: 53.4 months vs. 22.5 months) with DR, which also translated into a significant OS benefit (mOS: not reached vs. 47 months) [52]. Frontline regimens are summarized in Table 1.

**TABLE 1:** Main trial for first line setting.

Trial	Regimen	Number of patients	Median follow‐up	ORR	MRD‐negativity	PFS
CASSIOPIEA [35, 36]	DVTd versus VTd	543 versus 542	80.1 months	92.6% versus 89.9%	64% versus 44% (10^−5^)	Median: 83.7 versus 52.8 months
PERSEUS [37]	DVRd versus VRd	355 versus 354	47.5 months	96.6% versus 93.8%	75.2% versus 47.5% (10^−5^) 65.1% versus 32.2% (10^−6^)	48‐month PFS: 84.3% versus 67.7%
ISKIA [39]	IsaKRd versus KRd	151 versus 151	20 months	NA	77% versus 67% (10^−5^) 67% versus 48% (10^−6^)	NA
GMMG‐HD7 [38]	IsaVRd versus VRd	331 versus 329	4 years	86.4% versus 79%	66% versus 48% (10^−5^)	48‐months PFS: 76% versus 69%
MAIA [47, 53]	DRd versus Rd	368 versus 369	64.5 months	92.2% versus 81.6%	32.1% versus 11.1% (10^−5^)	Median: 61.9 versus 34.4 months
ALCYONE [49]	DVMP versus VMP	350 versus 356	74.7 months	90.9% versus 73.9%	28.3% versus 7.0% (10^−5^)	Median: 36.4 versus 19.3 months
IMROZ [38]	IsaVRd versus VRd	265 versus 181	69.7 months	NA	55.5% versus 40.9% (10^−5^)	Median: NR versus 54.3 months
CEPHEUS [51]	IsaVRd versus IsaRd	197 versus 198	58.7 months	97% versus 92%	60.9% versus 39.4% (10^−5^)	54‐months PFS: 68.1 versus 49.5 months
BENEFIT [50]	IsaRd versus IsaVRd	135 versus 135	23.5 months	78% versus 85%	26% versus 56% (10^−5^)	24‐months PFS: 80% versus 82.5%

Abbreviations: DRd, daratumumab‐lenalidomide‐dexamethasone; DVMP, daratumumab‐bortezomib‐melphalan‐prednisone; DVRd, daratumumab‐bortezomib‐lenalidomide‐dexamethasone; DVTd, daratumumab‐bortezomib‐thalidomide‐dexamethasone; IsaKRd, isatuximab‐carfilzomib‐lenalidomide‐dexamethasone; IsaVRd, isatuximab‐bortzezomib‐lenalidomide‐dexamethasone; KRd, carfilzomib‐lenalidomide‐dexamethasone; Rd, lenalidomide‐dexamethasone; VMP, bortezomib‐melphalan‐prednisone; VRd, bortezomib‐lenalidomide‐dexamethasone; VTd, bortezomib‐talidomide‐dexamethasone.

### 4.3 Relapsed/Refractory Setting

With the advent of new therapies, patients with NDMM are increasingly likely to be triple class exposed (to anti‐CD38 monoclonal antibodies, immunomodulatory drugs [IMiDs], and PIs) and become refractory to lenalidomide and anti‐CD38 agents after initial treatment. One of the major determinants of the treatment choice at relapse, is refractoriness to prior treatments. Consequently, second‐line treatment options (particularly for lenalidomide and/or bortezomib refractory patients) include pomalidomide (P)‐based, and carfilzomib‐based regimes, plus anti‐CD38‐antibodies or anti‐CD38‐free combinations depending on previous exposure and refractory status. In this context, a new very promising therapeutic approach involves regimens targeting B‐cell maturation antigen (BCMA). Relapsed/refractory regimens are summarized in Table 2.

**TABLE 2:** Main trial for relapsed setting.

Trial	Experimental arm schedule	Number of patients	Prior line	Median follow‐up	ORR	MRD‐negativity	PFS
ASPIRE [54, 55] (phase 3) KRd versus Rd	KRd: Lenalidomide 25 mg PO on days 1–21 of each cycle Carfilzomib 20 mg/m^2^ IV on days 1 and 2 of cycle 1; 27 mg/m^2^ on days 1, 2, 8, 9, 15, and 16 during cycles 1–12 followed by 27 mg/m^2^ on days 1, 2, 15, and 16 Dexamethasone 40 mg^a^ PO on days 1, 8, 15, and 22 28‐day cycle	396 versus 396	2 (1–3)	67 0.1 months	87.1% versus 66.7%	NA	26.3 versus 17.6 months
ENDEAVOR [56] (phase 3) Kd versus Vd	Kd: Carfilzomib 20 mg/m^2^ IV on days 1 and 2 of cycle 1 56 mg/m^2^ thereafter on days 1, 2, 8, 9, 15, and 16 of 28‐day cycles Dexamethasone 20 mg PO/IV on days 1, 2, 8, 9, 15, 16, 22, and 23 28‐day cycle	463 versus 456	2 (1–3)	12 months	76.9% versus 62.6%	NA	18.7 versus 9.4 months
POLLUX [57] (phase 3) DRd versus Rd	DRd: Daratumumab 16 mg/kg IV days 1, 8, 15 and 22 of cycles 1–2, and days 1 and 15 of cycles 3–6, and Q4W thereafter Dexamethasone 20 mg PO on days 1, 2, 8, 9, 15, 16 and 22, 23 28‐day cycle	286 versus 283	1 (1–11)	44 months	93% versus 72%	22.4% versus 4.6% (10^−5^)	44.5 versus 17.5 months
CASTOR [58] (phase 3) DVd versus Vd	DVd: Daratumumab 16 mg/kg IV days 1, 8, 15 in cycles 1–3, day 1 of cycles 4–8, then and day 1 (every 4 weeks) thereafter Bortezomib 1.3 mg/m^2^ SC on days 1, 4, 8, 11 until cycle 8 Dexamethasone 20 mg PO on days 1, 2, 4, 5, 8, 9, 11, 12 21‐day cycle (1–8) 28‐day cycle (9 onwards)	251 versus 247	2 (1–10)	40 months	85% versus 63%	11.6% versus 2.4% (10^−5^)	16.7 versus 7.1 months
ELOQUENT‐2 [59] (phase 3) Erd versus Rd	Erd: Elotuzumab 10 mg/kg IV days 1, 8, 15, and 22 of cycles 1–2 and then on days 1 and 15 cycle 3 thereafter; Lenalidomide 25 mg PO on days 1–21 of each cycle Dexamethasone 40 mg^a^ days 1, 8, 15, 22 per week^b^ 28‐day cycle	321 versus 325	2 (1–4)	48 months	79% versus 66%	NA	19.4 versus 14.9 months
TOURMALINE‐MM1 [60] (phase 3) XRd versus Rd	XRd: Ixazomib 4 mg PO days 1, 8, and 15 Lenalidomide 25 mg PO on days 1–21 of each cycle; dexamethasone 40 mg PO on days 1, 8, 15, and 22 28‐day cycle	360 versus 362	2 (1–3)	15 months	78% versus 71%	NA	20.6 versus 14.7 months
ELOQUENT‐3 [61] (phase 2) EPd versus Pd	EPd: Elotuzumab 10 mg/kg IV days 1, 8, 15, and 22, cycles 1–2 and 20 mg/kg on day 1 of each cycle thereafter Pomalidomide 4 mg PO on days 1–21 of each cycle Dexamethasone 40 mg^a^ days 1, 8, 15, 22 per week^b^ 28‐day cycle	60 versus 57	3 (2–8)	9 months	53% versus 26%	NA	10.3 versus 4.7 months
IKEMA [62, 63] (phase 3) IsaKd versus Kd	Isa‐Kd: Isatuximab 10 mg/kg EV weekly 4 weeks, and then every 2 weeks Carfilzomib 56–70 mg/m^2^ days 1, 8, and 15 (Cycle 1, day 1 dose is 20 mg/m^2^) Dexamethasone 40 mg OS/IV days 1, 8, 15, 22 28‐day cycle	179 versus 123	2 (1–3)	44 months	86.6% versus 83.7%	33.5% versus 15.4% (10^−5^)	35.7 versus 19.2 months
CANDOR [64, 65] (phase 3) DKd versus Kd	DKd: Daratumumab 1800 mg SC (or 16 mg/kg IV) days 1, 8, 15 and 22 of cycles 1–2, and days 1 and 15 of cycles 3–6, and Q4W thereafter Carfilzomib IV 56–70 mg/m^2^ days 1, 8, and 15 (Cycle 1, day 1 dose is 20 mg/m^2^) Dexamethasone 40 mg OS/IV days 1, 8, 15, 22 28‐day cycle	312 versus 154	2 (1–3)	50 months	84% versus 75%	28% versus 9% (10^−5^)	28.4 versus 15.2 months
OPTIMISMM [66] (phase 3) PVs versus Vd	PVd: Pomalidomide 4 mg PO days 1–14 Bortezomib SC 1.3 mg/m^2^ days 1, 4, 8, and 11 (cycles 1–8) and days 1 and 8 (> cycle 9) Dexamethasone 40 mg^a^ on days 1, 2, 4, 5, 8, 9, 11, and 12 (cycles 1–8) and on days 1, 2, 8, and 9 (cycle 9+) 21‐day cycle	281 versus 278	> 1	15.9 months	82.2% versus 50%	NA	11.2 versus 7.1 months
APOLLO [67] (phase 3) DPd versus Pd	DPd: Daratumumab 1800 mg SC days 1, 8, 15 in cycles 1–3, day 1 of cycles 4–8, then and day 1 28‐day cycle Pomalidomide 4 mg PO on days 1–21 of each cycle Dexamethasone 40 mg^a^ days 1, 8, 15, 22 per week^b^	151 versus 152	> 1	16.8 months	69% versus 46%	9% versus 2% (10^−5^)	12.4 versus 6.9 months
ICARIA [68, 69] (phase 3) IsaPd versus Pd	IsaPd: Isatuximab IV 10 mg/kg, days 1, 8, 15, and 22 in cycle 1; days 1 and 15 in subsequent cycles Pomalidomide 4 mg PO on days 1–21 of each cycle Dexamethasone 40 mg^a^ days 1, 8, 15, 22 per week^b^ 28‐day cycle	154 versus 153	≥ 2	52.4 months	60.4% versus 35.3%	6% versus 0 (10^−5^)	11.1 versus 5.9 months
BOSTON [70] (phase 3) SVd versus Vd	SVd: Selinexor 100 mg PO on days 1, 8, 15, 22, and 29 Bortezomib of 1.3 mg/m^2^ SC on days 1, 8, 15, and 22 Dexamethasone 20 mg PO dose on days 1, 2, 8, 9, 15, 16, 22, 23, 29, and 30 5‐week cycle	195 versus 207	2 (1–3)	13.2 months	76.4% versus 62.3%	5% versus 4% (10^−5^)	13.9 versus 9.4 months
KarMMa‐3 [71] (phase 3) Ide‐cell versus standard regimen (DPd, DVd, XRd, Kd, EPd)	Single ide‐cel infusion (target dose 150 × 10^6^ to 450 × 10^6^)	254 versus 132	2–4	18.6 months	71% versus 42%	20% versus 1% (10^−5^)	13.3 versus 4.4 months
CARTITUDE‐4 [72] Cilta‐cell versus standard regimen (PVd, DPd)	Single cilta‐cel infusion (target dose, 0.75 × 10^6^/m^2^)	208 versus 211	1–3	15.9 months	81% versus 46%	60% versus 15% (10^−5^)	11.8 months versus NR
DREAMM‐3 [73] (phase 2) Belantamab versus Pd	Belantamab mafodotin 2.5 mg/kg IV on day 1 21‐day cycle	218 versus 107	> 1	11.5 months	8% versus 25%	7% versus 0% (10^−5^)	11 versus 7 months
DREAMM‐7 [74] (phase 3) Bela‐Vd versus DVd	BVd: Belantamab mafodotin 2.5 mg/kg IV on day 1 Bortezomib of 1.3 mg/m^2^ SC on days 1, 4, 8, and 11 Dexamethasone PO/EV days 1, 2, 4, 5, 8, 9, 11, 12 21‐day cycle	243 versus 251	> 1	28.2 months	83% versus 71%	39% versus 17% (10^−5^)	37 versus 13 months
DREAMM‐8 [75] (phase 3) Bela‐Pd versus VPd	BPd: Belnatamab mafodotin 2.5 mg/m^2^ IV on day 1 of cycle 1 and 1.9 mg/m^2^ IV on day 1 of cycle 2 onward Pomalidomide 4 mg PO on days 1–21 of each cycle Dexamethasone 40 mg^a^ days 1, 8, 15, 22 per week^b^ 28‐day cycle	155 versus 147	> 1	21.8 months	77% versus 72%	32% versus 5% (10^−5^)	1‐year: 51% versus 71%
MAJESTECT‐1 [76] (phase 1/2) Teclistamab	1.5 mg/kg SC every week after receiving step‐up doses of 0.06 mg/kg and 0.3 mg/kg	165 (RP2D)	≥ 3	30.4 months	63%	29% (10^−5^)	11.4 months
MONUMENTAL‐1 [77] (phase 1/2) Talquetamab	0.4 mg/kg SC every week or 0.8 mg/kg every 2 weeks with step‐up doses	QW cohort: *n* = 143 Q2W cohort: *n* = 154; prior TCR cohort: *n* = 78	≥ 3	QW cohort: 29.8 months; Q2W cohort: 23.4 months; prior TCR cohort: 20.5 months	67%–74%	NA	7.5 versus 11.2 versus 7.7 months
MAGNETISMM‐3 [78] (phase 2) Elranatamab	76 mg SC once weekly in 28‐day cycles after two step‐up priming doses of 12 and 32 mg given on day 1 and day 4 of cycle 1^c^	123	5 (2–22)	14.7 months	61%	21% (10^−5^)	15‐month: 51%

Abbreviations: Bela‐Vd, belantamab mafodotin‐bortezomib‐dexamethasone; Cilta‐cel, ciltacabtagene autoleucel; DKd, daratumumab‐carfilzomib‐dexamethasone; DPd, daratumumab‐pomalidomide‐dexamethasone; DVd, daratumumab‐bortezomib‐dexamethasone; Erd, elotuzumab‐lenalidomide‐dexamethasone; Ide‐cel, idecabtagene vicleucel; IV, intravenously; Kd, carfilzomib‐dexamethasone; MRD, minimal residual disease; NA, not available; ORR, overall response rate; Pd, pomalidomide dexamethasone; PFS, progression‐free survival; PO, by mouth; PVd, pomalidomide‐bortezomib‐dexamethasone; Q2W, every 2 weeks; Q4W, every 4 weeks; QW, every week; RP2D, recommended phase 2 dose; SC, subcutaneously; SVd, selinexor‐bortezomib‐dexamethasone; TCR, triple class refractory; Vd, bortezomib‐dexamethasone; XRd, ixazomib‐lenalidomide‐dexamethasone.

^a^ In general, patients received oral dexamethasone at a dose of 40 mg (patients ≤ 75 years) or 20 mg (patients > 75 years).

^b^ In the days of elotuzumab administration, when patients in the elotuzumab group received dexamethasone both orally (28 mg in patients ≤ 75 years or 8 mg in patients > 75 years) and intravenously (8 mg).

^c^ After six cycles, persistent responders (partial response or better lasting at least 2 months) switched to a dosing interval of once every 2 weeks.

Belantamab mafodotin is a humanized anti‐BCMA antibody conjugated to monomethyl auristatin‐F, a microtubule‐disrupting agent. Over the past year, two recent Phase III studies, DREAMM‐7 [74] and DREAMM‐8 [75], have shown a significant PFS benefit of belantamab in combination with either bortezomib or pomalidomide, respectively, as a second‐line or later treatment. Main drug‐specific toxicity is corneal toxicity.

Idecabtagene vicleucel (ide‐cel) and ciltacabtagene autoleucel (cilta‐cel) are two distinct chimeric antigen receptor T cell (CAR‐T) products targeting BCMA, demonstrating high overall response rates (ORR) and prolonged PFS in their respective single arm clinical trials (CARTITUDE‐1 [79] and KarMMa‐1 [71]) in late lines. These products have also shown superior outcomes compared to standards of care, in early lines of therapy in the randomized CARTITUDE‐4 [80] and KarMMa‐3 [81] trials. EMA approved in 2024 cilta‐cel from second line and ide‐cel from third line in patients with triple class exposed disease, refractory to last line.

Two BCMA‐targeting bispecific antibodies, teclistamab [82] and elranatamab [78], and a bispecific antibody targeting G protein‐coupled receptor class C group 5 member D (GPRC5D), Talquetamab [83] have been approved for patients triple class exposed RRMM patients from fourth line.

Cytokine release syndrome, neurotoxicity, infections, and cytopenias are the most important adverse effects of both CAR‐T and bispecific antibodies [84]. With cilta‐cel, the potential for delayed neurotoxicity, including parkinsonian symptoms that may arise weeks or months after infusion, has been also reported [85]. Rate of infections with anti‐BCMA bispecific antibodies looks somehow higher than with CAR‐T cells anti‐BCMA, and bispecific antibodies against GPRC5D. Additionally, anti‐GPRC5D therapy has unique side effects, including dysgeusia, nail and skin toxicity, and weight loss, due to the on target of tumor activity [86].

Selinexor, selective inhibitor of nuclear export (SINE) that targets exportin 1 (XPO1), is also available in second line in combination with bortezomib and dexamethasone (SVd) [70], or in combination with dexamethasone alone (Sd) [87] in patients refractory to 2 PIs, 2 IMIDs and one anti CD38 monoclonal antibodies from fifth line.

### 4.4 Future Perspectives

New CAR‐T constructs and advanced manufacturing techniques are being explored to enhance efficacy, persistence, and accessibility. For example, anitocabtagene autoleucel (anito‐cel) is a BCMA‐directed CAR‐T therapy that incorporates a D‐domain binding motif, setting it apart from traditional BCMA CAR‐T therapies, which rely on single‐chain variable fragments for antigen recognition. In patients with a median of 4 lines of therapy, the ORR was 97% with good tolerability [88]. Conversely, arlocabtagene autoleucel (arlo‐cel) is an anti‐GPC5RD CAR‐T therapy that showed an ORR of 87% and a PFS of 18.3 months [89].

Still under investigation are Etentamig (ABBV‐383) (NCT06158841) and Linvoseltamab, two other bispecific antibodies targeting BCMAxCD3. A phase I/II trial of Linvoseltamab showed promising efficacy, with a 71% overall response rate (ORR) and 50% achieving complete response at the 200 mg dose. The median duration of response was 29.4 months [90].

As bispecific antibodies are very promising in heavily pretreated patients, ongoing trials are investigating these bispecific antibodies in earlier and different settings (e.g. in first‐line treatment [76], in maintenance [91]) or in combination therapies [92].

With a different target, Cevostamab, an FcRH5/CD3 bispecific antibody, has demonstrated clinical activity in heavily treated MM patients in a phase I trial [93]. Additionally, trispecific antibodies (ISB 2001–201) targeting BCMA, CD38, and CD3 have also shown preliminary promising results in patients who have previously received CD38 or BCMA therapy, with response rates ranging from 86% to 90% [94].

Among small molecules, novel cereblon E3 ligase modulatory drugs (CELMoDs), such as iberdomide and mezigdomide, are currently under clinical development [95, 96, 97].

## Conflicts of Interest

F.G. has received honoraria from AbbVie, Roche, Takeda, Pfizer, Sanofi, Celgene/Bristol Myers Squibb, Janssen, GlaxoSmithKline; has served on advisory board for Abbvie, Roche, Takeda, Pfizer, Sanofi, Celgene/Bristol Myers Squibb, Oncopeptides, Janssen¸ GlaxoSmithKline, Kyte, AstraZeneca. G.B. and E.M. have no conflict of interest.

## Peer Review

The peer review history for this article is available at https://www.webofscience.com/api/gateway/wos/peer-review/10.1002/hon.70067.

## Permission to Reproduce Material From Other Sources

No copyrighted material from other sources has been reproduced in this review.

## Data Availability

This review is based on previously published studies, all of which are publicly available and cited in the manuscript.
